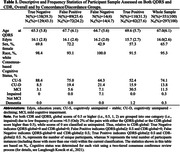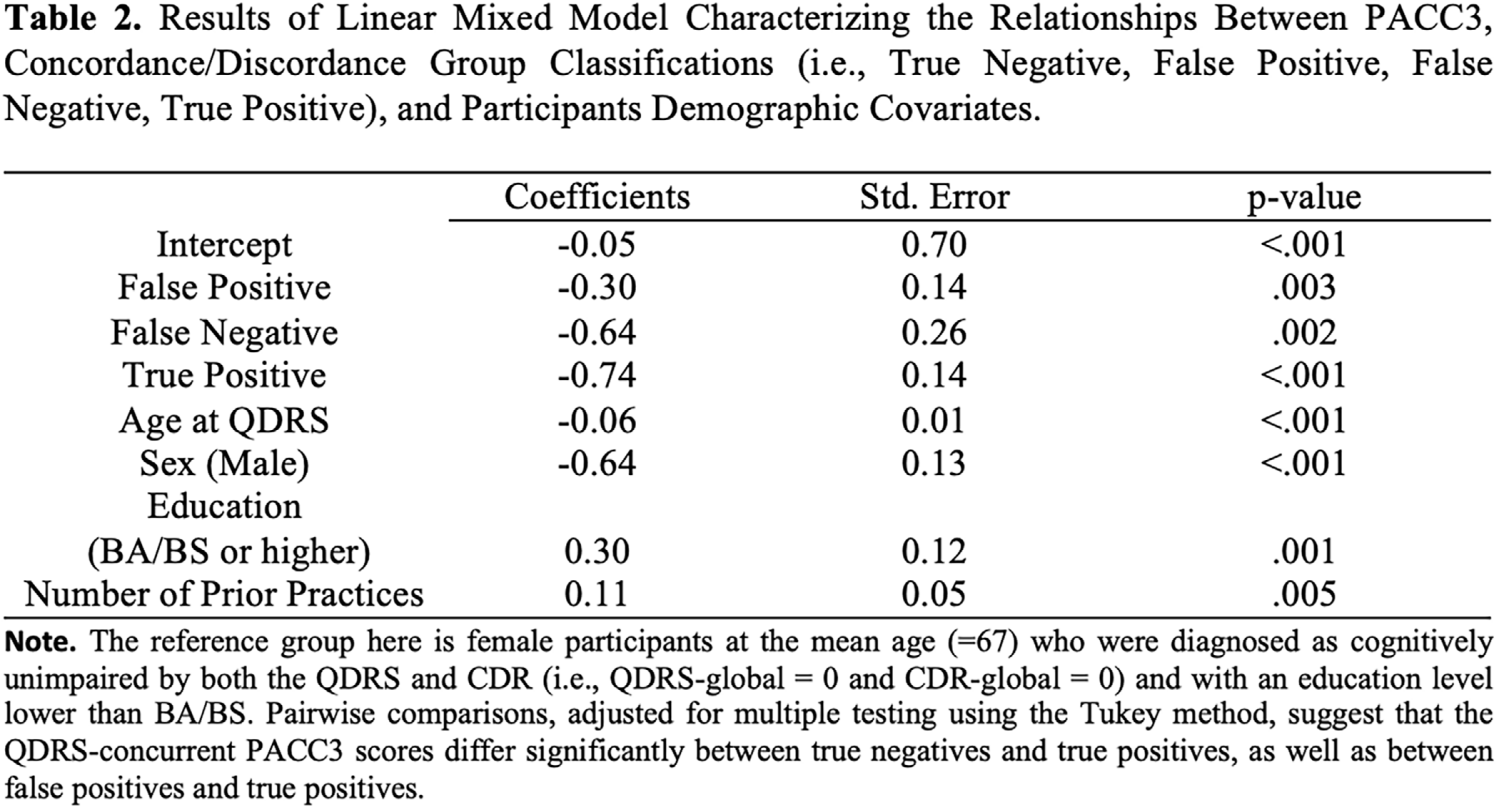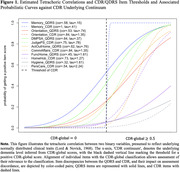# The Quick Dementia Rating System and the Clinical Dementia Rating Scale: Investigation of agreement and related features in a predominantly unimpaired sample

**DOI:** 10.1002/alz.090957

**Published:** 2025-01-03

**Authors:** Qi Huang, Daniel M. Bolt, Erin M. Jonaitis, Bruce P Hermann, Rachel L Studer, Brenda Ryther, Lia J. Sparks, James E Galvin, Sterling C. Johnson, Rebecca E. Langhough

**Affiliations:** ^1^ Department of Educational Psychology, School of Education, University of Wisconsin‐Madison, Madison, WI USA; ^2^ Wisconsin Alzheimer’s Institute, University of Wisconsin‐Madison School of Medicine and Public Health, Madison, WI USA; ^3^ Department of Medicine, University of Wisconsin‐Madison School of Medicine and Public Health, Madison, WI USA; ^4^ Wisconsin Alzheimer’s Disease Research Center, Madison, WI USA; ^5^ Department of Neurology, University of Wisconsin‐Madison School of Medicine and Public Health, Madison, WI USA; ^6^ University of Miami Miller School of Medicine, Boca Raton, FL USA; ^7^ Alzheimer’s Disease Research Center, University of Wisconsin‐Madison, Madison, WI USA; ^8^ Geriatric Research Education and Clinical Center, William S. Middleton Memorial Veterans Hospital, Madison, WI USA; ^9^ Wisconsin Alzheimer’s Institute, University of Wisconsin School of Medicine and Public Health, Madison, WI USA; ^10^ Wisconsin Alzheimer’s Disease Research Center, University of Wisconsin‐Madison School of Medicine and Public Health, Madison, WI USA; ^11^ Wisconsin Alzheimer’s Disease Research Center, University of Wisconsin School of Medicine and Public Health, Madison, WI USA

## Abstract

**Background:**

The Clinical Dementia Rating Scale (CDR) is a gold standard metric for staging the nature and severity of global cognitive and functional impairment in Alzheimer’s disease (AD) and other dementias. Prior evidence from older and/or smaller samples suggests that The Quick Dementia Rating System (QDRS) informant questionnaire provides results comparable to the CDR and can be completed in just 3‐5 minutes, sans a trained clinician or rater. This study aimed to: 1) investigate concordance between the QDRS‐derived global CDR (“QDRS‐global”; Galvin, 2015) and CDR‐global scores; 2) examine item‐level QDRS/CDR agreement; and (3) evaluate QDRS‐global/CDR‐global concordant/discordant groups against concurrent Preclinical Alzheimer’s Cognitive Composite (PACC3) performance.

**Method:**

The study included 351 QDRS/CDR pairs (n = 297 participants) from the Wisconsin Registry for Alzheimer’s Prevention (WRAP). Analyses include descriptive indices of QDRS/CDR agreement (e.g., QDRS‐global false negative [FN], false positive [FP] rates relative to CDR‐global scores (each collapsed to 0 = unimpaired;.5 = impaired due to few values>.5), Lasso logistic regression identifying QDRS items associated with discordance, tetrachoric correlations evaluating psychometric functioning of CDR and QDRS items, and linear mixed models examining associations between QDRS/CDR concordant/discordant groups and PACC3 performance.

**Result:**

The QDRS‐global/CDR‐global scores concordance rate was 70.66% (see also Table 1). Relative to CDR‐global, the QDRS‐global FP and FN rates were 39.2% and 11.3%, respectively. Item‐level results were consistent with this pattern for all item pairs, except for FuncHome/HomeHob showing a greater tendency for QDRS FNs. Logistic regression and psychometric analyses highlighted the QDRS Memory item as primarily responsible for FPs and FNs (Figure 1). After adjusting for covariates, QDRS‐concurrent PACC3 scores differed significantly for two pairs of QDRS‐global/CDR‐global concordance/discordance groups — True Negative (TN) vs. True Positive (TP), FP vs. TP (Table 2; Tukey‐adjusted pairwise comparisons). The latter suggests that QDRS‐global classification of impairment is more likely to be a FP‐misclassification for participants with high PACC3 scores than for those with low PACC3 scores.

**Conclusion:**

In this late‐middle‐aged, predominantly unimpaired sample, the QDRS appears well‐suited for screening for impairment. Future analyses will examine whether more nuanced scoring of the QDRS Memory item may reduce FP and FN rates and will investigate relations to biomarkers for AD and other dementias.